# Editorial: Clinical and molecular aspects of managing chronic urticaria: identifying endotypes, phenotypes, and factors determining responses and resistance to treatment

**DOI:** 10.3389/falgy.2026.1823731

**Published:** 2026-04-07

**Authors:** Andac Salman, Indrashis Podder, Jonny Peter, Ana Maria Gimenez-Arnau

**Affiliations:** 1 Department of Dermatology, Acibadem University School of Medicine, Istanbul, Turkiye; 2 Department of Dermatology, College of Medicine and Sagore Dutta Hospital, Kolkata, India; 3 Division of Allergy and Clinical Immunology, Department of Medicine, Groote Schuur Hospital, University of Cape Town, Cape Town, South Africa; 4 Allergy and Immunology Unit, University of Cape Town Lung Insitute, Cape Town, South Africa; 5 Department of Dermatology, Hospital del Mar Research Institute, Universitat Pompeu Fabra, Barcelona, Spain

**Keywords:** biomarker, chronic spontaneous urticaria (CSU), endotype, phenotype, precision medicine

Chronic spontaneous urticaria (CSU) is being increasingly recognized as a heterogeneous, immune-mediated disorder, not limited to mast cells alone. Advances in immunopathogenesis, biomarker research, and targeted therapies have reshaped the therapeutic landscape, taking us closer to precision medicine. A key prerequisite for precision medicine is the identification of relevant endotypes and phenotypes, aimed at predicting the therapeutic response and prognosis. The recently updated treatment guideline has expanded the therapeutic armamentarium to include newer biologics (remibrutinib and dupilumab) in addition to omalizumab, further necessitating patient stratification strategies (1). The current research Topic in *Frontiers in Allergy*, titled “*Clinical and molecular aspects of managing Chronic Urticaria: Identifying endotypes, phenotypes, and factors determining responses and resistance to treatment,”* aimed to contribute to the understanding of current personalized treatment strategies in CSU based on endotypes, phenotypes, and other clinically relevant factors.

Su Küçük and Yücel provide a comprehensive overview of CSU immunological endotypes, distinguishing type I (autoallergy) and type IIb (autoimmune) CSU, along with overlapping and mixed forms. They highlight the role of IgE autoantibodies, IgG anti-Fc*ε*RI/IgE antibodies, complement activation, coagulation pathways, and the emerging evidence of MRGPRX2-mediated mast cell activation. They focus on readily available laboratory markers—total serum IgE, eosinophil and basophil counts, inflammatory markers, thyroid autoantibodies, and the autologous serum skin test (ASST) to stratify patients into endotypes. An endotype-oriented treatment approach is proposed for antihistamine refractory CSU, type I endotype favouring omalizumab and type IIb endotype favouring omalizumab or cyclosporine. Although many of these markers are pending validation, the proposed endotype-oriented treatment algorithm is a starting point for individualized management of CSU, especially when refractory to antihistamines.

Despite the introduction of newer therapeutic options like remibrutinib and dupilumab in recent urticaria guideline, second-generation antihistamines (sgAHs) and omalizumab remain the 1st and 2nd line treatment modalities in step-wise algorithmic approach. However, many patients remain poorly controlled, highlighting the need for predictors of treatment response. In this context, Calzari et al. review predictors of response to second-generation antihistamines (sgAHs) and omalizumab. Elevated CRP or Il-6, high baseline disease activity, concomitant chronic inducible urticaria (CIndU), high neutrophil-lymphocyte ratio, high D-dimer and fibrinogen (coagulation markers), eosinopenia, and basopenia predict poor response to antihistamines, supporting earlier treatment escalation in selected patients. Regarding omalizumab, factors correlating with reduced response include advanced age, obesity, autoimmune comorbidities, low total IgE (<40–50 IU/ml), positivity for ANA or anti-TPO antibodies and basophil activation marker 203c. High total IgE levels and elevated basophil FcɛRI expression remain the strongest predictor of favourable response to omalizumab. Thus, a composite immunological signature involving multiple biomarkers is key to predict therapeutic response and facilitate patient-centric management in CSU.

Mateu-Arrom et al. further consolidate this precision framework by exploring predictors of omalizumab response and relapse following discontinuation. Signatures of type IIb autoimmunity including low total IgE level, high CRP, basopenia, eosinopenia and anti-TPO positivity correlate with poor response. Concomitant inducible urticaria may warrant longer duration of omalizumab therapy, while patients with higher BMI may need higher doses. Higher baseline disease activity and longer disease duration indicate increased recurrence risk following omalizumab withdrawal. The authors suggest that gradual tapering following complete symptom control may mitigate relapse in high-risk individuals. This review highlights clinically accessible parameters to predict omalizumab response and relapse risk following discontinuation, thus enabling individual treatment decisions and counselling in regular practice. However, further large-scale studies are needed to validate these findings.

Beyond treatment predictors, recent research is focussing on pathogenic heterogeneity in CSU, unravelling novel mechanistic insights to facilitate targeted therapies. In their systematic review and meta-analysis, Xue et al. evaluate Th1-, Th2-, and Th17-related cytokines, identifying significant elevations in TNF-α and IL-17 levels in CSU patients, compared to age-and-sex matched healthy controls. These findings question the type-2 dominance in CSU pathogenesis and highlight the contribution of non–type 2 pathways, particularly Th17-associated inflammation. However, heterogeneity across studies and variability in assay methodology limit immediate clinical translation. This review suggests to expand the diagnostic work-up of CSU to include non-Th2 cytokines as well, paving the way for pathway-specific interventions in near future.

Comorbidity screening is important in CSU patients as they may influence disease course and treatment response. Atopic comorbidities such as atopic dermatitis (AD), allergic rhinitis and asthma often co-exist in such patients as they share a common pathogenic pathway. This notion is further strengthened as Toubi et al. report higher frequency of AD (10%) in their cohort of CSU patients (*n* = 425). CSU patients with concomitant AD had higher frequency of asthma, elevated serum IgE level and more severe disease, compared to those without comorbidities. Dupilumab provided substantial benefit to a subset of omalizumab refractory CSU + AD patients, suggesting common pathogenic signature. These authors concluded that AD may co-exist in CSU patients, because of Th2 dominance in both disorders. High total IgE and asthma may indicate an underlying AD component in CSU patients, which may necessitate T-cell directed biologic therapies if conventional therapies remain ineffective.

Lebedkina et al. further broaden the phenotypic spectrum of chronic urticaria (CU), who report that patients with mixed [>1] chronic inducible urticaria (CIndU) have significantly higher frequency of concomitant CSU (*p* < 0.001) and angioedema (*p* = 0.014), compared to those with standalone CIndU (*n* = 188, 125-standalone CIndU, 63- mixed CIndU). Mixed CIndU patients have more comorbid CSU, higher total serum IgE, but lower rates of allergic diseases such as AD. The authors propose that patients with multiple inducible subtypes may represent a subgroup with heightened mast cell reactivity or systemic susceptibility. So, the combination of >1 CIndUs (mixed) may represent a distinct phenotype requiring special attention from treating physicians.

In summary, this research topic collection highlights several important aspects in the current landscape of CSU pathogenesis and management. It stresses the fact that immunological endotyping, biomarker exploration, therapeutic prediction, comorbidity screening and phenotypic characterization are crucial for personalized care of CSU patients, instead of a one-size-fits-all approach, which is often inadequate. This would greatly improve the treatment outcome in CU patients, by providing long-term disease control and improving their quality of life. However, these concepts need further validation by multi-centre, large-scale longitudinal cohorts involving heterogeneous CU patients, to generate robust real-world data and integrate them into routine clinical practice.
